# Changes in THC Positivity Rates in Adolescents Corresponding to Legalization of Recreational Marijuana in Illinois

**DOI:** 10.5811/westjem.62946

**Published:** 2026-05-19

**Authors:** Eriq Gassé, April Brill

**Affiliations:** Midwestern University – Chicago College of Osteopathic Medicine, Department of Emergency Medicine, Downers Grove, Illinois

## Abstract

**Introduction:**

As public access to tetrahydrocannabinol (THC) products expands under state legalization, there is concern for increased use in adolescents under 18 years of age and its subsequent effects. Adverse effects of THC use have been documented such as epilepsy, lethargy, somnolence, and respiratory insufficiency. Additional concern has been raised regarding whether potential increases in THC intoxications presented to the emergency department (ED) would demand a simultaneous increase in resource utilization, like lab testing and hospital admission. Previous studies on the relationship between marijuana and pediatric use demonstrated mixed results, with others indicating an increase in co-ingestion rates when pediatric patients use marijuana.

**Objective:**

To investigate whether the incidence of THC use in pediatrics increased post-recreational marijuana legalization in 2020 (RML) and if the incidence of co-ingestion in these cases differed significantly from the pre-RML data.

**Methods:**

This was a retrospective cohort study examining positive urine drug screens (UDS) in pediatrics during an emergency department visit at a community hospital during fixed time periods in 2019 and 2020, on either side of Illinois’ legalization of recreational marijuana on January 1st, 2020. Pediatric subjects were defined as those younger than 18 years of age and older than four weeks of age at the time of UDS collection with valid, complete, and reported laboratory results. UDS screens analyzed in this study were collected in the ED of a suburban community hospital from pediatric charts housed on the electronic medical record, where there was a clinical indication for UDS collection and regardless of disposition. UDS may have been obtained for either medical or psychiatric concerns, but the reason for the UDS or patient visit was not collected or analyzed. UDS was considered positive if either amphetamines, benzodiazepines, opiates, Phencyclidine (PCP), cocaine, or barbiturates were identified as present in the urine sample and negative if none of these substances were detected in the urine sample. Both negative and positive UDS were assessed using chi-square and odds ratios to investigate these differences and compare across sex.

**Results:**

There were 169 patients with a mean age of 14 years included. Mean age in 2019 (14.1 ± 3.2 years) was comparable to mean age in 2020 (14.7 ± 2.0 years) (two-sample t-test: 0.143 > 0.05). Sex demographics were also not significantly different between cohorts, with 53.4% being female in 2019 and 50.6% in 2020 (chi-square: 0.1318; significance: 0.1318 > 0.05). Between the two time periods, we found no significant difference in pediatric presentation to the ED with THC positive testing (2019: 16 positive UDS of 88 collected [18.18%]; 2020: 17 positive UDS of 81 collected [20.99%]; chi square: 0.211; Significance: 0.646 > 0.05; odd ratio between group: 1.195 [CI 95%: 0.558–2.559]) nor correlation with demographic data. We found a nonsignificant positive association between pediatric THC ingestion and co-ingestion of other substances (amphetamines, benzodiazepines, opiates and cocaine) pre-RML and a nonsignificant negative association post-RML (2019: 1.092 [CI 95%: 0.742–1.606]; 2020: 0.913 [CI 95%: 0.627–1.330]).

**Conclusion:**

These findings indicate that THC usage among pediatric patients with positive UDS did not increase with state legalization, nor did co-ingestion rates. There was also no difference in rates of positive or negative UDS between patient sex. This must be considered clinically when anticipating potential co-ingestions while interacting with children who may be using street drugs. Additionally, this data suggests that legalization of recreational marijuana does not significantly change the rate of children presenting to the ED with positive drug screens and can be used by hospital administration and state representatives to anticipate healthcare demands following large-scale drug reclassifications. Further investigation is warranted to evaluate these findings in a larger, multi-center study.

